# Supplementary material for: A geometric-surface PDE model for cell-nucleus translocation through confinement
Source: PLoS Comput Biol. 2026 Jul 30;22(7):e1014569. doi: 10.1371/journal.pcbi.1014569 (PMC13456492; doi:10.1371/journal.pcbi.1014569)
Supplement: Supplementary file 1 [file pcbi.1014569.s003.pdf]

## S1 Text

We model the cell surface using a tensorial Jeffreys fluid model, as done in [1] and for which we have the mechanical data. Rheologically, this is represented by a 1D circuit composed of two branches connected in series: a Kelvin-Voigt element (Branch 1) and a pure viscous dashpot (Branch 2), as described in Fig. SF1.

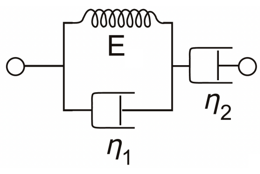


Figure SF1: Jeffrey's model described as 1D circuit composed of two branches connected in series: a Kelvin-Voigt element (Branch 1) and a pure viscous dashpot (Branch 2).

Because the elements are in series, the total surface stress tensor $\boldsymbol{\sigma}$ is uniform across both branches ($\boldsymbol{\sigma} = \boldsymbol{\sigma}_1 = \boldsymbol{\sigma}_2$), while the total surface rate-of-strain tensor $\mathbb{D}$ is the sum of the individual strain rates ($\mathbb{D} = \mathbb{D}_1 + \mathbb{D}_2$).

Branch 1 (the Kelvin-Voigt element) consists of a spring with elastic modulus $E$ and a dashpot with viscosity $\eta_1$ arranged in parallel. The stress in this branch is the sum of the elastic and viscous contributions:

$$\boldsymbol{\sigma} = 2E\boldsymbol{\epsilon} + 2\eta_1\mathbb{D}_1\,, \tag{1}$$

where $\boldsymbol{\epsilon}$ is the surface strain tensor such that its objective time derivative yields the rate-of-strain ($\partial_t\boldsymbol{\epsilon} = \mathbb{D}_1$).

In Branch 2 (the purely viscous dashpot), the stress is proportional to its rate-of-strain:

$$\boldsymbol{\sigma} = 2\eta_2\mathbb{D}_2\,, \tag{2}$$

where $\eta_2$ is the viscosity of the pure fluid component.

Taking the time derivative of the stress in Branch 1 (1) yields:

$$\partial_t\boldsymbol{\sigma} = 2E\mathbb{D}_1 + 2\eta_1\partial_t\mathbb{D}_1\,. \tag{3}$$

To obtain a single constitutive equation for the total stress, we substitute the kinematic compatibility condition $\mathbb{D}_1 = \mathbb{D} - \mathbb{D}_2$ into Equation (3):

$$\partial_t\boldsymbol{\sigma} = 2E(\mathbb{D} - \mathbb{D}_2) + 2\eta_1\partial_t(\mathbb{D} - \mathbb{D}_2)\,. \tag{4}$$

From the Branch 2 constitutive law (2), we know that $\mathbb{D}_2 = \frac{\boldsymbol{\sigma}}{2\eta_2}$. Substituting this into (4):

$$\partial_t\boldsymbol{\sigma} = 2E\mathbb{D} - \frac{E}{\eta_2}\boldsymbol{\sigma} + 2\eta_1\partial_t\mathbb{D} - \frac{\eta_1}{\eta_2}\partial_t\boldsymbol{\sigma}\,. \tag{5}$$

Rearranging the terms, we obtain:

$$\boldsymbol{\sigma} + \left(\frac{\eta_1 + \eta_2}{E}\right)\partial_t\boldsymbol{\sigma} = 2\eta_2\mathbb{D} + 2\frac{\eta_1\eta_2}{E}\partial_t\mathbb{D}\,. \tag{6}$$

From a computational standpoint, computing the time derivative of the rate-of-strain ($\partial_t \mathbb{D}$) is numerically challenging because it requires tracking second-order time derivatives of the surface position. To elegantly bypass this, we decompose the total stress into an instantaneous viscous part ($2\eta_s \mathbb{D}$) and a memory-dependent polymeric part ($\boldsymbol{\sigma}_p$)[2, 3]:

$$\boldsymbol{\sigma} = 2\eta_s \mathbb{D} + \boldsymbol{\sigma}_p \,, \tag{7}$$

where we strategically define the effective instantaneous viscosity as $\eta_s = \frac{\eta_1 \eta_2}{\eta_1 + \eta_2}$. Substituting this decomposition into Equation 6 yields:

$$2\eta_s \mathbb{D} + \boldsymbol{\sigma}_p + \left(\frac{\eta_1 + \eta_2}{E}\right)(2\eta_s \partial_t \mathbb{D} + \partial_t \boldsymbol{\sigma}_p) = 2\eta_2 \mathbb{D} + 2\frac{\eta_1 \eta_2}{E} \partial_t \mathbb{D} \,. \tag{8}$$

The terms involving $\partial_t \mathbb{D}$ perfectly cancel out on both sides of the equation. This isolation provides a clean, and easily implementable evolution equation for the polymeric stress $\boldsymbol{\sigma}_p$:

$$\boldsymbol{\sigma}_p + \left(\frac{\eta_1 + \eta_2}{E}\right) \partial_t \boldsymbol{\sigma}_p = 2\frac{\eta_2^2}{\eta_1 + \eta_2} \mathbb{D} \,. \tag{9}$$

Then, we arrive at the final constitutive law governing the viscoelastic relaxation:

$$\boldsymbol{\sigma}_p + \tau_p \partial_t \boldsymbol{\sigma}_p = 2\eta_p \mathbb{D} \,, \tag{10}$$

where $\tau_p = \frac{\eta_1 + \eta_2}{E}$ is the relaxation time and $\eta_p = \frac{\eta_2^2}{\eta_1 + \eta_2}$ is the effective polymeric viscosity.

Finally, in the case one wants to model the cell as a 2D cross-section, the active surface reduces to a 1D closed contour. Consequently, the tensorial quantities defined on the manifold naturally simplify to their scalar counterparts along the curve. The polymeric stress tensor $\boldsymbol{\sigma}_p$ reduces to the scalar macroscopic line tension $T_p$, while the rate-of-strain tensor $\mathbb{D}$ simplifies to $VH$, where $V$ and $H$ are the normal velocity and the curvature of the contour, respectively. The final constitutive law governing the viscoelastic relaxation thus becomes the scalar evolution equation:

$$T_p + \tau_p \partial_t T_p = 2\eta_p V H \,. \tag{11}$$

# References


[1] Jebane C, Varlet AA, Karnat M, Cedillo L, Lecchi A, Bedu F, et al. Enhanced cell viscosity: A new phenotype associated with lamin A/C alterations. iScience. 2023 08;26:107714. doi:10.1016/j.isci.2023.107714.

[2] Rajagopalan D, Armstrong RC, Brown RA. Calculation of steady viscoelastic flow using a multimode Maxwell model: application of the explicitly elliptic momentum equation (EEME) formulation. Journal of Non-Newtonian Fluid Mechanics. 1990;36:135-57. doi:10.1016/0377-0257(90)85007-L.

[3] Salbreux G, Jülicher F. Mechanics of active surfaces. Physical Review E. 2017 Sep;96:032404. doi:10.1103/PhysRevE.96.032404.
